# Evaluation of the effects of different stocking densities on the sediment microbial community of juvenile hybrid grouper (♀*Epinephelus fuscoguttatus* × ♂ *Epinephelus lanceolatus*) in recirculating aquaculture systems

**DOI:** 10.1371/journal.pone.0208544

**Published:** 2018-12-20

**Authors:** Xiaoyan Chen, Tianyun Shao, Xiaohua Long

**Affiliations:** Jiangsu Provincial Key Laboratory of Marine Biology, College of Resources and Environmental Sciences, Nanjing Agricultural University, Nanjing, P.R. China; University of Trento, ITALY

## Abstract

Aquatic microorganisms are an important part of aquatic ecosystems because they are involved in nutrient cycling and water quality, eventually influencing fish productivity. However, at present, reports on the effect of stocking density on microorganisms in sediment samples in recirculating aquaculture systems (RAS) are relatively rare. In this study, the changes in the microbial community in an RAS were investigated under different stocking densities of juvenile hybrid grouper (♀*Epinephelus fuscoguttatus* × ♂*Epinephelus lanceolatus*). Total DNA was extracted from the sediment samples, the 16S rDNA gene was amplified, and the bacterial community was analysed by Illumina high-throughput sequencing. We identified 741 OTUs from a total of 409,031 reads. Based on the analysis of bacterial composition, richness, diversity, bacterial 16S and rDNA gene abundance; sediment sample comparisons; and the existence of specific bacterial taxa within four densities, we concluded that the dominant phyla in all samples were similar and included: Proteobacteria, Bacteroidetes, Nitrospirae, Planctomycetes, Verrucomicrobia, and Chloroflexi. However, their relative distributions differed at different fish densities. Linear discriminant analysis further indicated that the stocking treatment influenced the sediment bacterial community. This study indicates that under RAS aquaculture, mode density is a factor regulating the microbial community, which provides insights into microbe management in RAS culture.

## Introduction

Currently, aquatic food production has transitioned from being primarily based on the capture of wild fish to culturing an increasing number of farmed species. A milestone was reached in 2014 when the aquaculture sector’s contribution to the supply of fish for human consumption overtook that of wild-caught fish for the first time. China, with more than 60% of the global aquaculture production [1], has played a major role in this growth. Generally, grouper is a popular food fish, and its production has been steadily rising with increasing global demand. Therefore, the sustainable aquaculture of grouper and related species deserves further development. China, a major exporter of grouper, has great potential for market development supplying a rapidly growing middle class [2]. Through the development of hybridization technology, scientists crossed giant grouper (*Epinephelus lanceolatus*) males with tiger grouper (*Epinephelus fuscoguttatus*) females to produce a new variety of grouper, which is morphologically similar to its parental species [3].

Recirculating aquaculture systems (RAS) in intensive aquaculture production have become important production systems due to the potential to reduce water and energy consumption and create a stable cultivation tank environment [4]. These systems require a high initial capital investment for the construction of farms, as well as intensive management, but this is matched by the high output due to high stocking density. However, an excessively high stocking density may be associated with growth, health and fish welfare problems [5,6] and variations in the microbial communities in the water and sediment, including the development of pathogens [7].

Aquatic microorganisms are an important part of aquatic ecosystems because they are involved in nutrient cycling and water quality, eventually influencing fish productivity [8]. The diversity of microbial communities can change the aquaculture ecosystem and reflect the state of the aquaculture environment. A large number of researches on the environmental microorganisms of aquatic ecosystems had shown that various types of microorganisms have a variety of functions: Some play an important role in the process of material transformation, such as participating in the carbon and nitrogen cycle and the transformation of material energy [9,10]. At the same time, it helps to reduce the content of ammonia-nitrogen waste in water bodies and ensure the quality of water bodies [11,12]. Some may directly biodegrade some exogenous organic pollutants [13,14,15,16]. Aquatic ecosystems include bacteria, archaea, fungi, and probiotics. The analysis of sediment bacteria information can help advance the knowledge of the bacteria-aquaculture system interactions.

Therefore, the aim of this study was to use 16S rDNA gene sequencing to characterize bacterial communities in the sediment of an RAS environment supporting different stock densities of hybrid grouper. This knowledge will provide a theoretical basis for further improving the quality of water bodies as well as aquatic products.

## Materials and methods

### Ethics statement

The all of the procedures involving fish were carried out in accordance with the Guidelines for the Care and Use of Laboratory Animals prepared by the Institutional Animal Care and Use Committee of Nanjing Agricultural University, Nanjing, China (SYXK(SU)2017–0007). And the Institutional Care and Use Committee (IACUC) specifically approved this study. The experimentation was performed in the laboratory animal center of Nanjing Agricultural University.

### Sampling procedure

The experiment was carried out in an RAS with uniform specifications in the Laboratory of the College of Resources and Environmental Sciences, Nanjing Agricultural University (Nanjing, China). Using the flow model, inlet pipe is made by 45° bend PVC tube to form the lateral jet, a circular sump pit is installed in the center of the bottom of the pond. A circular sump pit is set up at the center of the pool bottom, equipped with PVC sewage discharge device, in order to remove the bottom center of the fish tank residue, feces and fish tank surface oil film. The fish were provided by Qingdao General Aquaculture Co., Ltd.. The hybrid groupers (average body mass = 25.43 ± 2.36 g) were cultured at four treatment densities with three replicates: low stocking density, 1.03 kg m^−3^ (LD); medium stocking density, 2.06 kg m^−3^ (MD); high stocking density, 3.09 kg m^−3^ (HD); and extra-high stocking density, 4.11 kg m^−3^ (EHD). All treatments were randomly assigned to triplicate tanks (diameter = 80 cm, depth = 70 cm) for a period of 22 weeks (19 November 2015 to 28 April 2016). During the experimental period, the mean temperature was 23.1 ± 2.7°C, the photoperiod was 10 h light: 14 h dark (natural lighting), the salinity was 25 g L^-1^, NH_3_-N 0.01–0.05 mg L^-1^, NO_2_-N 0.05–0.10 mg L^-1^, and the pH was 7.5 ± 0.3. Dissolved oxygen remained above 5 mg L^-1^ with continuous aeration. Flow rate was about 150–180 L/h. Depending on the water quality conditions, approximately 5–10% of the water was exchanged weekly. The main nutrient components in fish diet (purchased from Tianjin Haifa Treasures Industrial Development Co.) were as follows (in % w/w): crude protein ≥ 50.0, crude fat ≥ 8.0, crude fiber ≤ 2.0, crude ash ≤ 15.0, total phosphorus 0.5–2.2, total calcium 1.3–3.0, water ≤ 10.0). All groupers were hand-fed slowly on formula feed twice daily (9:00 and 18:00). The daily feed rations were adjusted based on weight of the fish (approximately 2–2.5% of fish weight•day^-1^). No food residues were observed on the bottom of the tank after feeding procedures. No antibiotic treatments were used at any time. At harvest, the feed conversion ratio (FCR), condition factor (K, %) and specific growth rate (SGR, % day^−1^) were calculated as below:

$$\begin{array}{l} FCR=\frac{W_{f}}{W_{t}‑W_{0}}\\ K(\%)=\frac{W_{t}}{L_{3}}\times 100\\ SGR(\%\cdot day^{-1})=\frac{\ln W_{t}-\ln W_{0}}{t}\times 100 \end{array}$$

where W_0_ = initial body mass (g); W_t_ = final body mass (g); W_f_ = total food intake (g); L = total length (cm); and t = sampling time (days)

On the final day of the experiment (22 weeks), the sediments attached to the surface of the container samples were collected from three places in each tank (water depth was 50 cm). An equivalence mixture was made using the slurry sediment samples from each tank’s culture (approximately 5 g) and was stored at -80°C until further analysis.

### Extraction and sequencing of bacterial 16S rDNA

In each tank, 1 g of sediment sample was used for DNA extraction. The bacterial DNA was extracted with a PowerSoil DNA Isolation Kit (MO BIO Laboratories, Inc, Carlsbad, CA). The 16S rDNA variable V3-V4 region was amplified with the primer pair 338F (5'-ACTCCTACGGGAGGCAGCA-3') and 806R (5'-GGACTACHVGGGTWTCTAAT-3'). The PCR was performed at TransGen AP221-02 with TransStart FastPfu DNA polymerase (TransGen Biotech, Beijing, China). The 50 μL PCR mixture was composed of 5 μL 10× Pyrobest buffer, 4 μL dNTPs, 2 μL of each forward and reverse primers, 0.3 μL Pyrobest DNA polymerase (2.5 U μL^-1^), 30 ng of the DNA template and ddH_2_O. After an initial denaturation at 95°C for 5 min, 25 cycles of 30 s at 95°C, 30 s at 56°C and 40 s at 72°C were performed. The final extension step consisted of 10 min at 72°C. All the amplifications were checked using electrophoresis with 2% w/w agarose gels. The bands were extracted and purified with the AxyPrepDNA gel (Axygen, CA, USA). Pyrosequencing of the amplifications was performed on the Illumina MiSeq platform (Beijing Allwegene Technology Inc., Beijing, China). The complete data sets were deposited in NCBI under GenBank accession number PRJNA392047.

### Bioinformatics analysis

To estimate the alpha diversity, an operational taxonomic unit (OTU) table was rarified, and four metrics were calculated: Chao 1 to estimate richness, observed OTUs to identify the unique OTUs found in the sample, and the Shannon and Simpson indices to estimate the diversity [17].

Based on the 16S rDNA PCR amplification, linear discriminant analysis (LDA) combined with effect size (LEfSe) analysis was conducted to identify species with significant differences in abundance among treatment groups and to construct cladograms [18]. The clustering analysis of samples (hierarchical clustering at the phylum level) was conducted by using the unweighted pair group method with arithmetic means [19].

### Statistical analyses

The statistical analyses were performed using SPSS (version 19.0) software. Normality and homogeneity of variances were checked by the Kolmogorov–Smirnov and Levene tests, respectively. The data were analysed by one-way analysis of variance (ANOVA) and were presented as the means ± standard errors. Tukey's test was used to compare differences among means (p ≤ 0.05).

A redundancy discrimination analysis (RDA) was used to characterize bacterial abundance and diversity in relation to fish physiological explanatory variables using Vegan 2.3.0, a package of R functions used for community ecology.

## Results

### Hybrid groupers culture results

**Fig 1:**
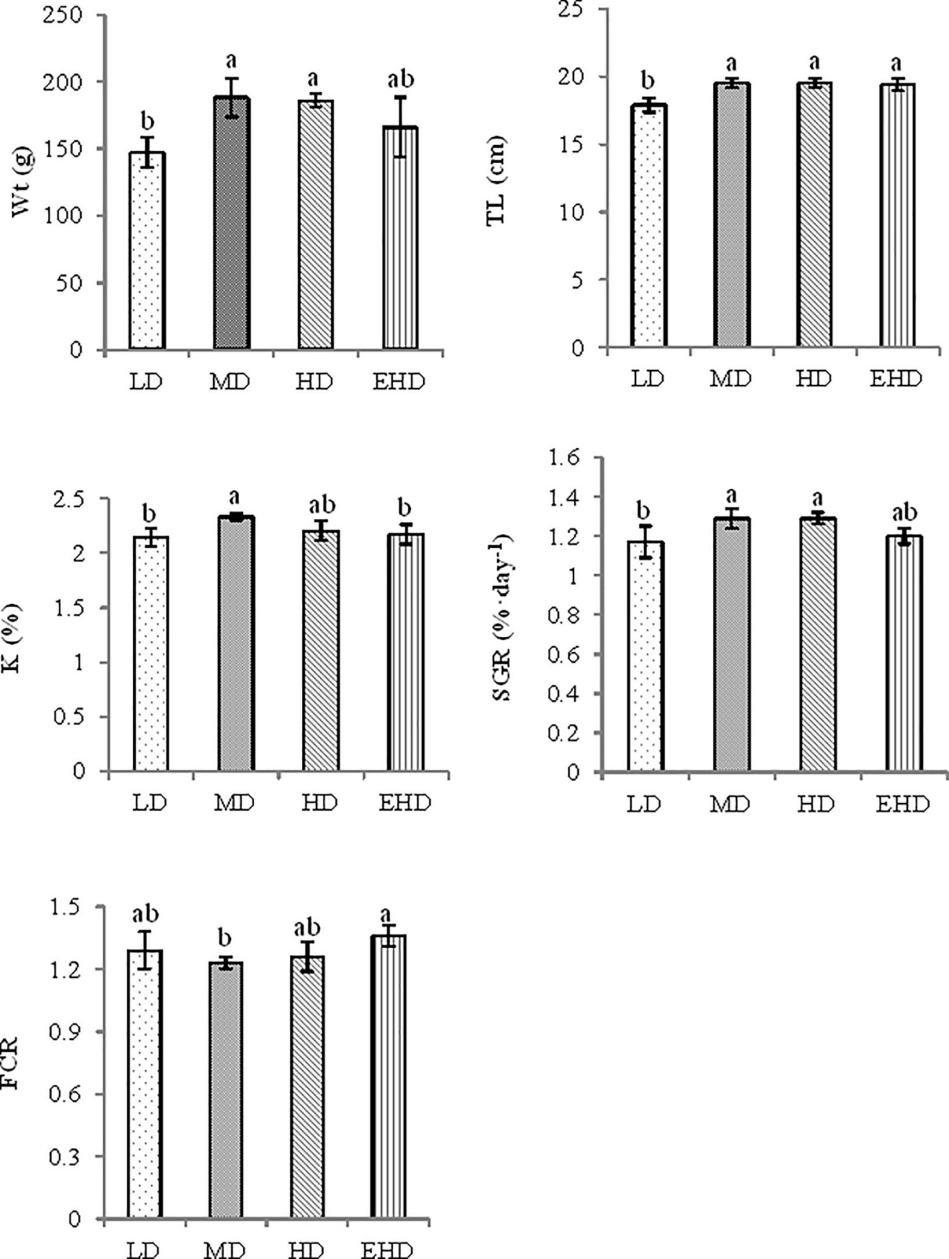
Growth and feeding indicesas influenced by different stocking densities of hybrid groupers. Note: LD—low stocking density; MD—middle stocking density; HD—high stocking density; EHD—extra high stocking density. Wt—final weight; TL—total length: K—condition factor; SGR—specific growth rate; FCR—feed conversion rate. Different letters in a single organ indicated significant differences among the experimental groups (P ≤ 0.05). Data are means ± S.E.M. The lowercase letters in the table represent significant differences between the different treatments in each index.

The final grouper weight and specific growth rate were greater at medium and high density compared with low density, but there was no significant difference between the low and extra high stocking densities (Fig 1). The fish length was significantly shorter at LD group compared with all other treatments (p < 0.05). Under extra high stocking density, the impacts were shown at a significantly higher condition factor (K, %) than under a medium density, whereas the feed conversion rate was the reverse (extra high was higher than a medium stocking density).

### Bacterial community composition in the sediments

**Fig 2:**
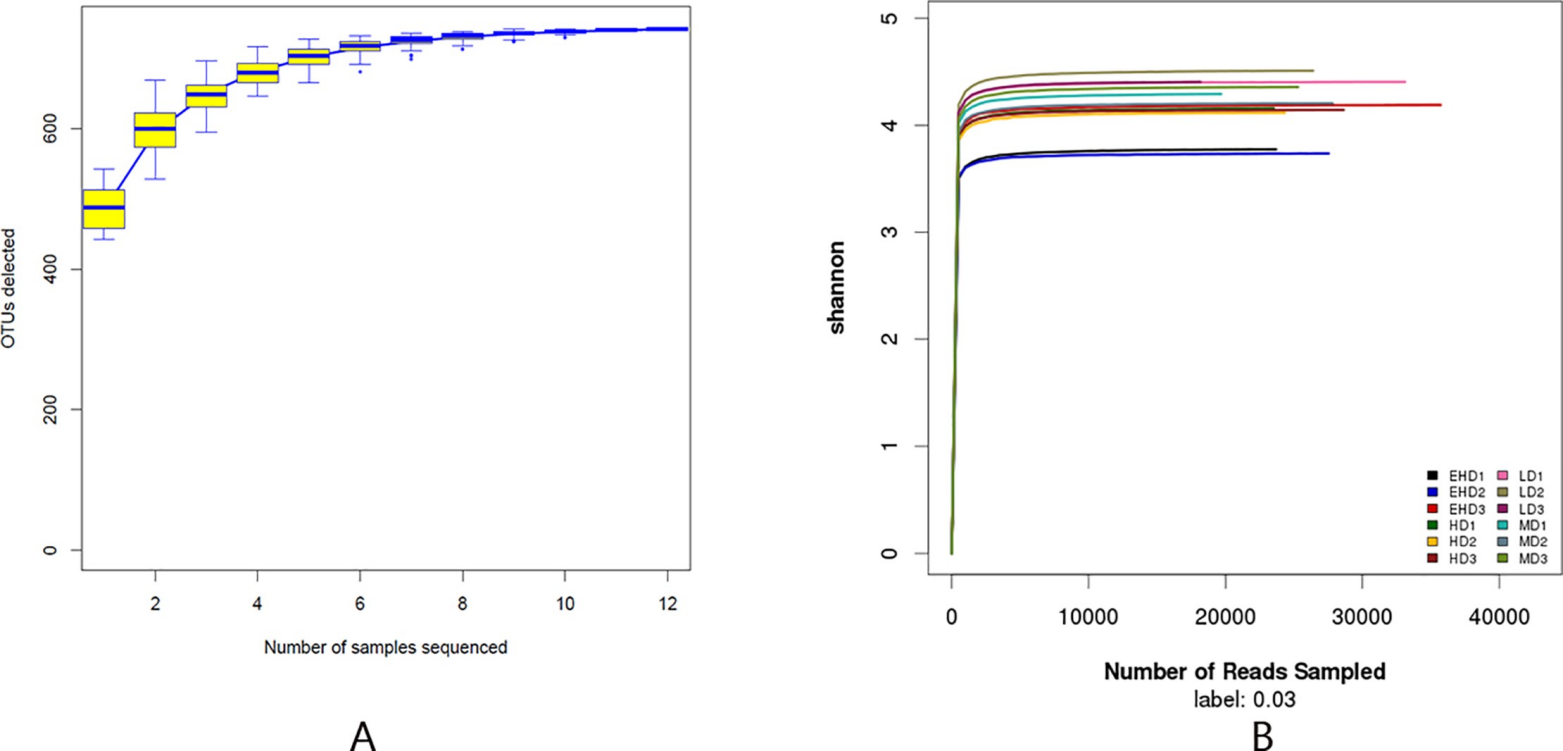
Species accumulation curves (A) and Shannon-Wiener curves (B) in the sediment of treatments with different stocking densities of hybrid groupers. Note: LD—low stocking density; MD—middle stocking density; HD—high stocking density; EHD—extra high stocking density.

**Fig 3:**
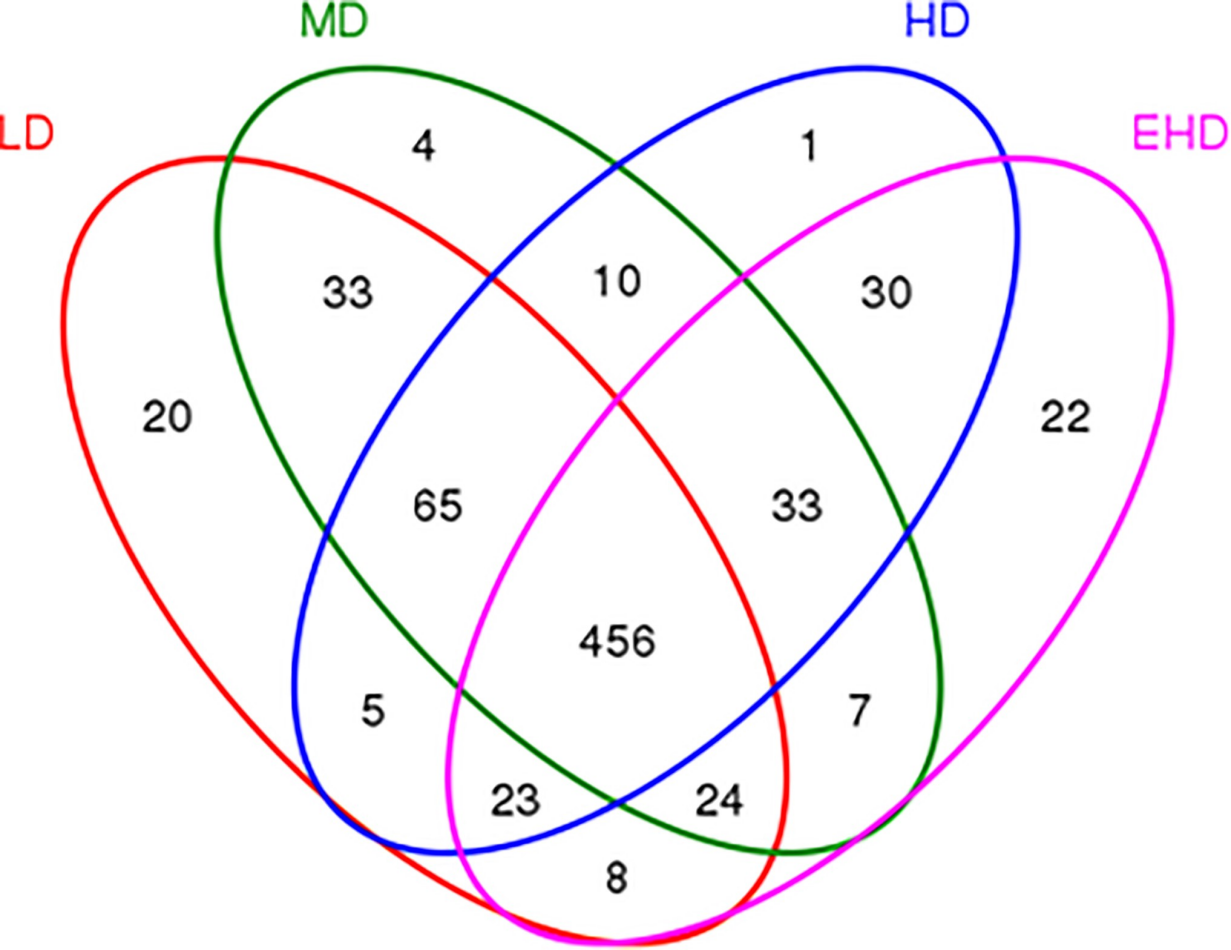
Venn of bacterial communities (based on OTUs at 3% distance) in the sediment of treatments with different stocking densities in hybrid groupers. Note: LD—low stocking density; MD—middle stocking density; HD—high stocking density; EHD—extra high stocking density.

Sequencing of the 12 sediments yielded a total of 409,031 sequences, of which 99.99% of the sequence length was in the range of 360–480 bp. Species accumulation curves (Fig 2A) and Shannon-Wiener curves (Fig 2B) indicated the number of samples and the sequencing depth was sufficient [20]. At 97% gene similarity, a total of 635, 633, 623 and 602 OTUs were identified in the LD, MD, HD and EHD groups, respectively. The Venn diagrams are shown in Fig 3. The number of unique OTUs in each density group was 20(LD), 4(MD), 1(HD) and 21(EHD), respectively. Bacterial richness and diversity are shown in Table 1. It can be deduced that the abundance and diversity of microbial communities were both decreasing as density increases.

**Table 1:** The alpha diversity indices of bacterial communities (based on OTUs at 3% distance) in the sediment of treatments with different stocking density of hybrid grouper individuals.

	Chao1 Index	Shannon Index	Simpson Index	Observed_species Index
LD	562±18^a^	6.40±0.05^a^	0.03±0.00^b^	466.67±13.50^a^
MD	563±16^a^	6.17±0.06^ab^	0.03±0.00^b^	444.13±11.80^ab^
HD	532±30^a^	5.96±0.02^bc^	0.04±0.00^b^	422.53±18.50^ab^
EHD	512±11^a^	5.62±0.21^c^	0.06±0.01^a^	406.63±15.06^b^

Note: LD—low stocking density; MD—middle stocking density; HD—high stocking density; EHD—extra high stocking density. The lowercase letters in the table represent significant differences between the different treatments in each index.

**Fig 4:**
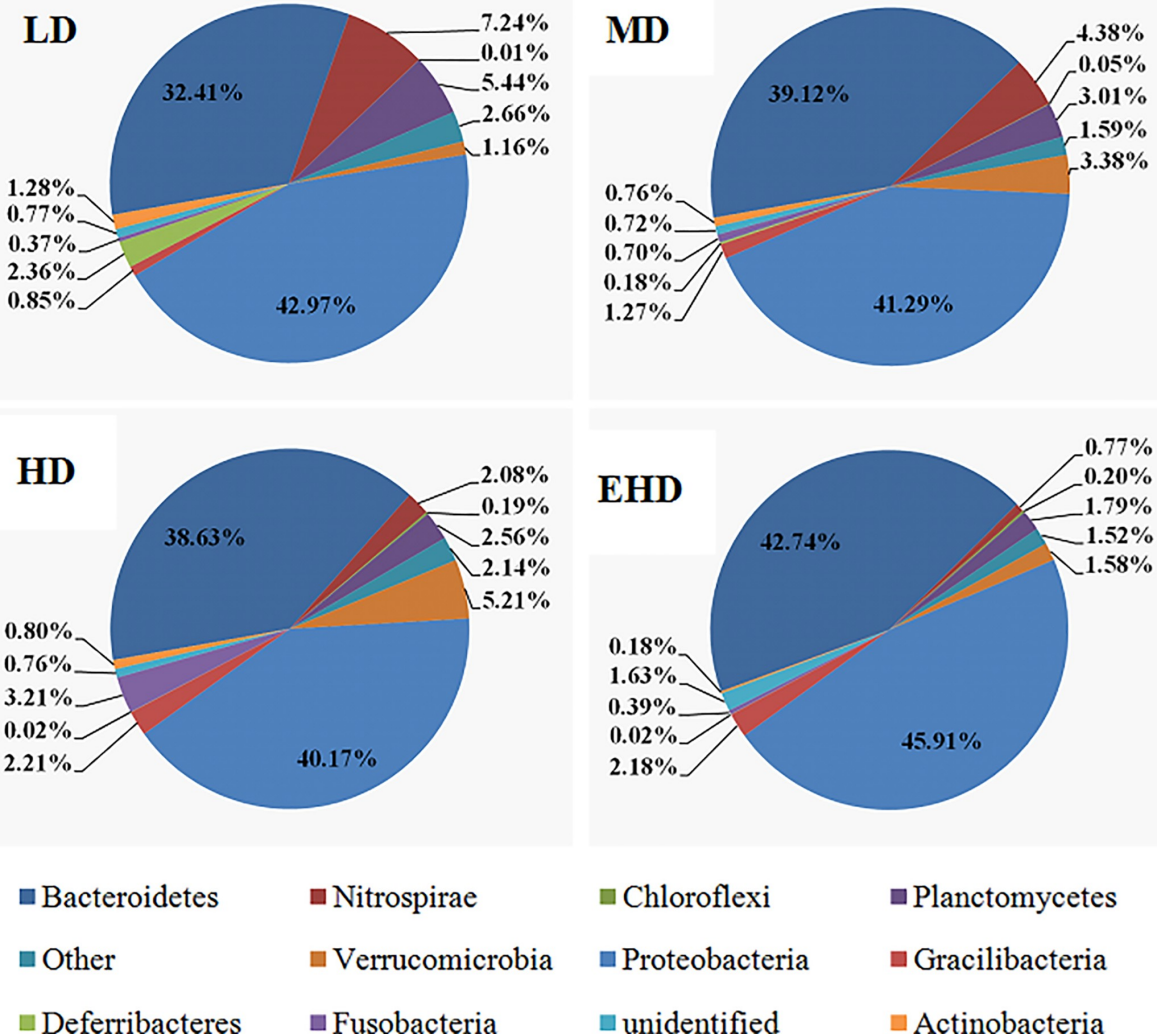
The relative abundance of 12 phyla in the sediment from treatments with different stocking densities of hybrid groupers. Note: LD—low stocking density; MD—middle stocking density; HD—high stocking density; EHD—extra high stocking density.

Overall, these OTUs were classified into 28 phyla indicating taxonomically diverse microbial communities at all samples. At this level, the dominant phyla were Proteobacteria and Bacteroidetes, which shared 75.58% - 88.38% of the total reads, while the other OTUs were mainly classified to Nitrospirae, Planctomycetes, Verrucomicrobia, Chloroflexi, Gracilibacteria, Fusobacteria, unidentified, Actinobacteria and Deferribacteres. Among them, Nitrospirae, Planctomycetes, Chloroflexi and Actinobacteria significantly decreased in abundance with an increase in density. The remainder of the phyla abundance was rare with a sum of 1.52–2.66% (Fig 4).

**Fig 5:**
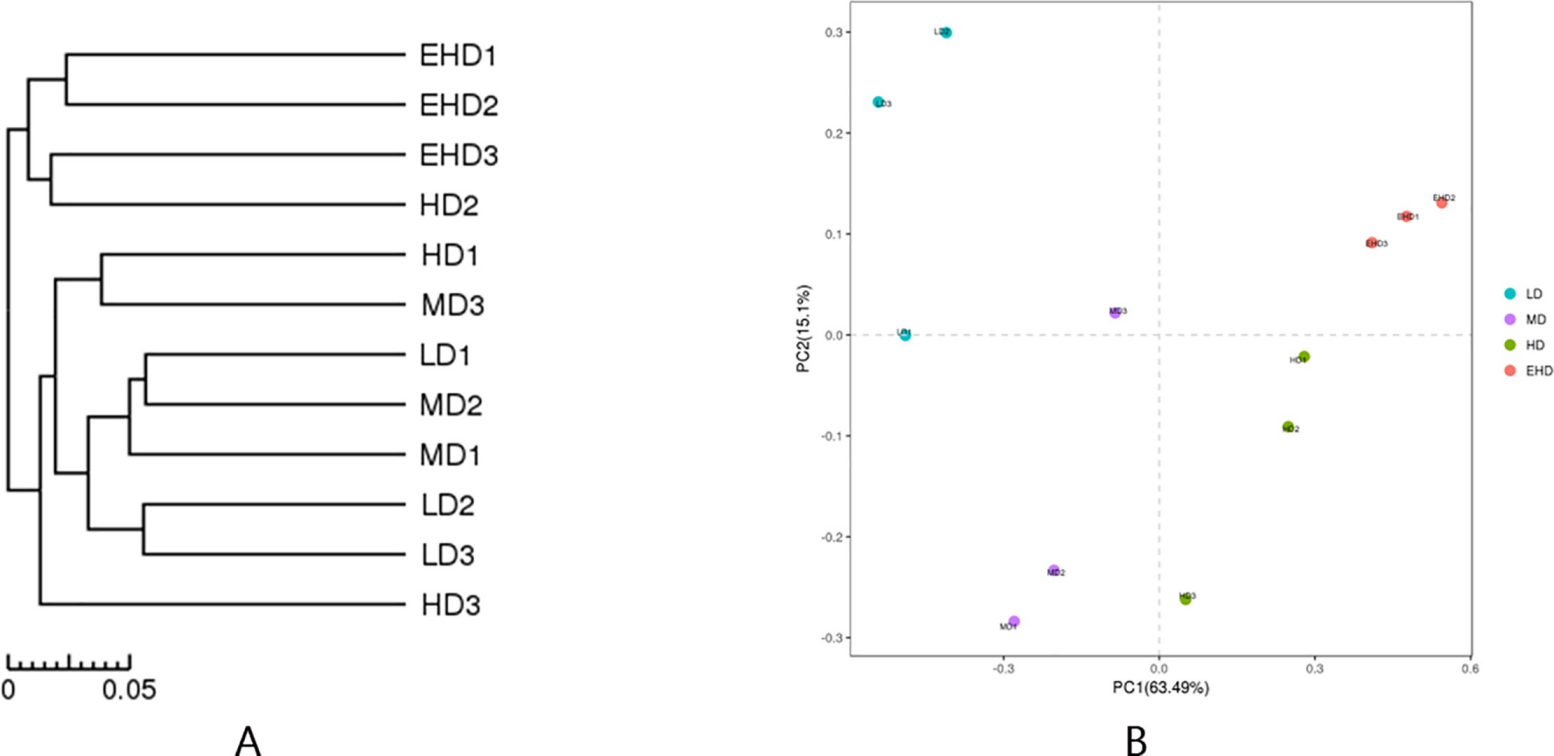
Cluster analysis (A) and PCA analysis (B) in the sediment from treatments with different stocking densities of hybrid groupers. Note: LD—low stocking density; MD—middle stocking density; HD—high stocking density; EHD—extra high stocking density.

Through a cluster analysis at the phylum level (Fig 5A), we found that only samples from EHD groups clustered together. While the sample of the remaining three groups had no obvious cluster trend, the general trend was adjacent density groups were similar to the sample communities. Principal component analysis results (Fig 5B) also showed that three samples in the EHD group were closely clustered together, indicating that the highest stocking density further reduces the dispersion of the sample.

**Fig 6:**
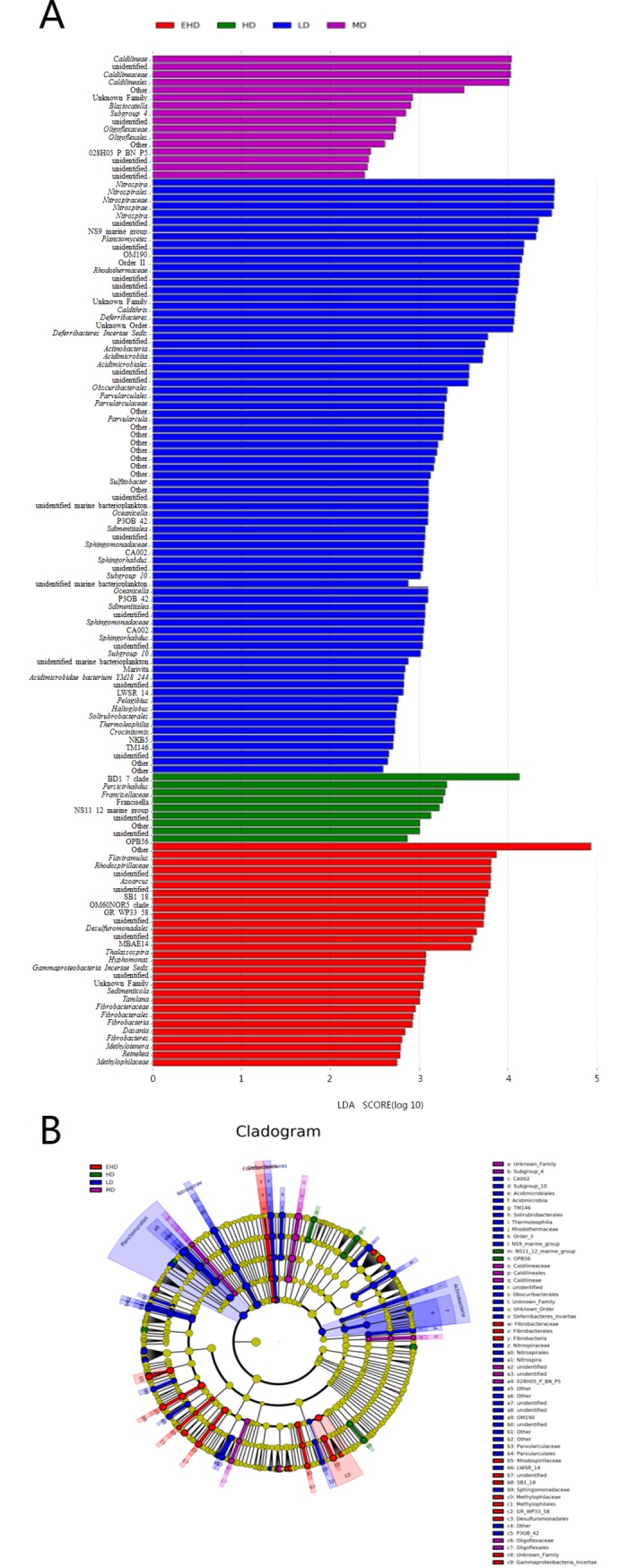
LDA EffectSize (LEfSe) first provided the list of features that differed among the treatments, ranking them according to the effect size (A); Differences were then mapped to taxonomic trees (B). Note: LD—low stocking density; MD—middle stocking density; HD—high stocking density; EHD—extra high stocking density. In the cladogram, the taxa highlighted by small circles and shading in the color of a specific treatment played an important role in the microbial community in that treatment; yellow color represented taxa that did not play an important role in microbial communities.

The distribution histograms of LDA scores (Fig 6A) showed that species had significantly different abundances among different stocking densities, with the length of the column representing the magnitude of the species influence. The cladograms indicate the taxa (highlighted by small circles and shading) that played important roles in the microbial community (Fig 6B).

Microbial phyla with greater abundance at low density compared to others were Nitrospirae, Planctomycetes, Deferribacteres and Actinobacteria. While Fibrobacteres had an extra high density. The following microbial families were more abundant: NS9_marine_group, Rhodothermaceae, P3OB-42, Sphingomonadaceae, LWSR-14 and Parvularculaceae in LD; Caldilineaceae, Blastocatella and Oligoflexaceae in MD; Persicirhabdus, Francisellaceae, NS11-12_marine_group and OPB56 in HD; and Flaviramulus, Rhodospirillaceae and Azoarcusin EHD.

### Redundancy discrimination analysis (RDA)

**Fig 7:**
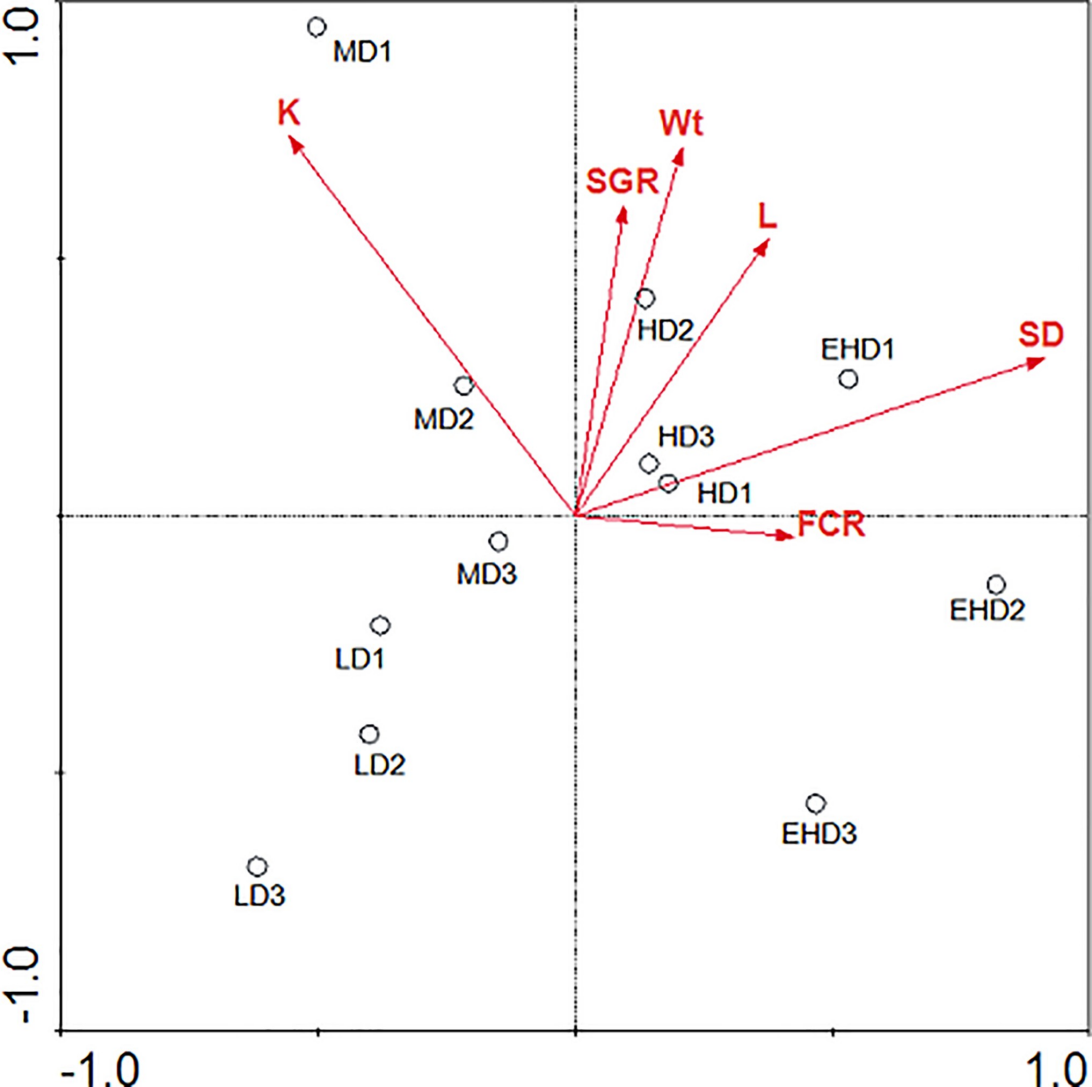
Redundancy analysis (RDA) of bacterial community structure in the sediment treatments from different stocking densities of hybrid groupers. Note: LD—low stocking density; MD—middle stocking density; HD—high stocking density; EHD—extra high stocking density. K—condition factor; FCR—feed conversion ratio; SGR—specific growth rate; Wt -final weight; L—total length; SD- stock density.

RDA was conducted to analyse the diversity distribution of bacterial communities under the four densities in response to grouper growth status (Fig 7). RDA analysis indicated that the K and SD (stocking densities) levels were most strongly associated with bacterial community diversity in the four tested environments.

## Discussion

There is widespread recognition that inappropriate stocking density is one of the main causes of poor fish growth and survival [21]. Considering all the factors (Wt, TL, K, SGR and FCR), the results indicated that acclimation of juveniles to the middle density conditions (between 2.06 and 3.09 kg m^−3^) enhanced more growth with less food consumption. These findings suggest that low and extra high stocking density might alter growth in hybrid groupers. The best growth was achieved in the MD and HD treatments. For example, higher mass gain, K, SGR and lower FCR are in line with the previous reports [22,23,24,25].

Given the importance of microbes in marine ecosystems, one of the key questions of microbial ecology is how microbial communities respond to anthropogenic activities. Microbial communities drive organic matter decomposition and nutrient cycling [26]. With the increase of stocking density, the total number of OTUs in sediments has decreased significantly, which is the same as change of bacteria diversity. However, the numbers of unique OTUs in the LD and EHD groups were more than in the MD and HD groups, perhaps related to the physicochemical properties of sediments in these two more specific environments (In the sediment samples, the TN was 0.78±0.14, 0.99±0.25, 1.17±0.22 and 1.32±0.41 g kg^-1^, while the TP was 1.03±0.26, 1.34±0.17, 1.35±0.34 and 1.53±0.2 g kg^-1^, in the LD, MD, HD and EHD samples respectively). The lower Chao 1, Shannon and higher Simpson indexes for the bacterial communities in EHD compared to other samples indicated that high density decreased the diversity and abundance of bacteria in sediment.

Previous studies on marine microbial communities showed that Proteobacteria and Bacteroidetes were dominant groups in seawater at different seasons all around the world [27,28,29]. In fact, in freshwater habitats, many reports on bacterial communities in the sediments of rivers, lakes, and reservoirs have shown similar results [30,31,32]. In this study, our results showed that the first dominant phylum is Proteobacteria (40.17–45.91%), and there was no significant difference in abundance between the four groups. Proteobacteria generally function in sulfate reduction, sulfur oxidization and nitrate assimilation [33,34]. The second dominant phylum, Bacteroidetes, was in a higher proportion in the sediment column of EHD, which is widely distributed in marine waters and has the potential of degrading biopolymers [35,36]. Additionally, we found that the abundance of Nitrospirae came in third place, with very few similarities with previous reports. The possible reason was that the RAS farming model differs from others and its aquaculture water is highly mobile. However, between different densities, the difference was significant in LD (7.24%) and EHD (0.77%). The same trend of negatively correlated abundances with density, also appeared in Planctomycetes, Chloroflexi and Actinobacteria. The common characteristics of these bacteria are that they play important roles in cycling of sulfur, nitrogen and organic compounds in marine sediment [37,38,39,40] and may be correlated with high organic content in the HD and EHD sediments.

Past studies generally described the relationship between the bacterial composition and abiotic environments, such as pH, temperature and ammonia. However, in this experiment, we focused on the relationship between bacterial composition and biological factors. According to the RDA analysis, K and SD were the most influential factors controlling the bacterial community diversity in our sediment, and the three factors of SGR, Wt and L were highly correlated. Contrary to the results of Fan [41], FCR was not a decisive factor for bacterial growth, and a possible reason was the circulation of water in this farming model. Considering that K and SD were significantly affected by the aquaculture space, these results reveal that aquaculture space should be a factor regulating the structure of the bacterial community. Certainly, bacteria diversity could also vary with sediment oxygen values by selecting for anoxic versus aerobic bacteria strains, which should be studied in the future.

In conclusion, 635, 633, 623 and 602 OTUs were identified from the sediment in the LD, MD, HD and EHD groups, respectively. Bacterial diversity and stability were higher in the LD than in the EHD. Bacterial composition in the sediment between different stocking densities were similar at the phylum level but varied with content. In addition, the bacterial communities showed clear responses to differences, including different K and different stocking densities.

## References

[1] FAO. The State of World Fisheries and Aquaculture. Contributing to food security and nutrition for all. Rome. 2016; 0–200.

[2] Stéphanie P, Sandrine G, Nathalie PD, Josiane A, Odile RC, Daniel LT, et al Grouper aquaculture: Asian success and Mediterranean trials. Aquat Conserv. 2008; 18: 297–308. 10.1002/aqc.840

[3] Ch’ng CL, Senoo S. Egg and larval development of a new hybrid grouper, tiger grouper Epinephelus fuscoguttatus × giant grouper E. lanceolatus. Aquac Sci. 2008; 56: 505–512.

[4] Summerfelt ST, Vinci BJ. Better management practices for recirculating aquaculture systems Environmental Best Management Practices for Aquaculture (eds Tucker C. S and Hargreaves J. A.), Wiley-Blackwell, Oxford, UK, 2008; 389–426.

[5] Castillo-Vargasmachuca S, Ponce-Palafox JT, García-Ulloa M, Arredondo-Figueroa JL, Ruiz-Luna A, Chávez EA, et al Effect of stocking density on growth performance and yield of subadult Pacific red snapper cultured in floating sea cages. Aquaculture. 2012; 74: 413–418. 10.1080/15222055.2012.676002

[6] Ni M, Wen HS, Li JF, Chi ML, Bu Y, Ren YY, et al The physiological performance immune responses of juvenile Amur sturgeon (Acipenser schrenckii) to stocking density and hypoxia stress. Fish Shellfish Immun. 2014; 36: 325–335. 10.1016/j.fsi.2013.12.002 24355406

[7] Kautsky N, Ronnback P, Tedengren M, Troell M. Ecosystem perspectives on management of disease in shrimp pond farming. Aquaculture. 2000; 191: 145–161. 10.1016/S0044-8486(00)00424-5

[8] Moriarty DJW. The role of microorganisms in aquaculture ponds. Aquaculture. 1997; 151: 333–349. 10.1016/S0044-8486(96)01487-1

[9] Cotner JB, Biddanda BA. Small players, large role: Microbial influence on biogeochemical processes in pelagic aquatic ecosystems. Ecosystems. 2002; 5(2): 105–121. 10.1007/s10021-001-0059-3

[10] Falkowski PG, Fenchel T, Delong EF. The microbial engines that drive Earth's biogeochemical cycles. Science. 2008; 320(5879): 1034–1039. 10.1126/science.1153213 18497287

[11] Fonseca AC, Summers RS, Hernandez MT. Comparative measurements of microbial activity in drinking water biofilters. Water Res. 2001; 35(16): 3817–3824. 10.1016/S0043-1354(01)00104-X 12230164

[12] Yu X, Shi X, Wei B, Ye L, Zhang S. PLFA profiles of drinking water biofilters with different acetate and glucose loadings. Ecotoxicology 2009; 18(6): 700–706. 10.1007/s10646-009-0346-x 19507021

[13] Chinalia FA, Killham KS. 2,4-dichlorophenoxyacetic acid (2,4-D) biodegradation in river sediments of Northeast-Scotland and its effect on the microbial communities (PLFA and DGGE). Chemosphere. 2006; 64(10): 1675–1683. 10.1016/j.chemosphere.2006.01.022 16488464

[14] Mannisto MK, Salkinoja-Salonen MS, Puhakka JA. In situ polychlorophenol bioremediation potential of the indigenous bacterial community of boreal groundwater. Water Res. 2001; 35(10): 2496–2504. 10.1016/S0043-1354(00)00527-3 11394785

[15] Slater GF, Cowie BR, Harper N, Droppo IG. Variation in PAH inputs and microbial community in surface sediments of Hamilton Harbour: Implications to remediation and monitoring. Environ Pollut. 2008; 153(1): 60–70. 10.1016/j.envpol.2007.08.009 17920174

[16] Bull AT, Stach JE, Ward AC, Goodfellow M. Marine actinobacteria: Perspectives, challenges, future directions. Anton Leeuw Int J G. 2005; 87(3): 65–79. 10.1007/s10482-004-6562-8 15971359

[17] Tatiana AV, Jennifer JM, Anthony VP, Zamin KY, Mircea P, Steven DB, et al Mercury and other heavy metals influence bacterial community structure in low-order Tennessee streams. Appl Environ Microb. 2011; 77(1): 302–311. 10.1128/AEM.01715-10 21057024 PMC3019708

[18] Nicola S, Jacques I, Levi W, Dirk G, Larisa M, Wendy SG, et al Metagenomic biomarker discovery and explanation. Genome Biol. 2011; 12(6): R60 10.1186/gb-2011-12-6-r60 PMC3218848 21702898

[19] Jiang XT, Peng X, Deng GH, Sheng HF, Wang Y, Zhou HW, et al Illumina Sequencing of 16S rRNA Tag Revealed Spatial Variations of Bacterial Communities in a Mangrove Wetland. Microb Ecol. 2013; 66: 96–104. 10.1007/s00248-013-0238-8 23649297

[20] Edgar RC. Search and clustering orders of magnitude faster than BLAST. Bioinformatics 2010; 26(19): 2460–2461. 10.1093/bioinformatics/btq461 20709691

[21] Barton BA. Stress in fishes: a diversity of responses with particular reference to changes in circulating corticosteroids. Integr Comp Biol. 2002; 42: 517–525. 10.1093/icb/42.3.517 21708747

[22] Eroldogan OT, Kumlu M, Aktas M. Optimum feeding rates for European sea bass Dicentrarchuslabrax L. reared in seawater and freshwater. Aquaculture. 2004; 231: 501–515. 10.1016/j.aquaculture.2003.10.020

[23] Sobrino I, Baldo F, Garcia-Gonzalez D, Cuesta JA, Silva-Garcia A, Fernandez-Delgado C, et al The effect of estuarine fisheries on juvenile fish observed within the Guadalquivir estuary (SW Spain). Fish Res. 2005; 76: 229–242. 10.1016/j.fishres.2005.06.016

[24] Tiago A, António A, Amalia Pérez-Jiménez, Aires Oliva-Teles, Verónica Heras, Juan MM, et al Evaluation of different stocking densities in a Senegalese sole (Solea senegalensis) farm: implications for growth, humoral immune parameters and oxidative status. Aquaculture. 2015; 438, 6–11. 10.1016/j.aquaculture.2014.12.034

[25] Bolasina S, Tagawa M, Yamashita Y, Tanaka M. Effect of stocking density on growth, digestive enzyme activity and cortisol level in larvae and juveniles of Japanese flounder, Paralichthysolivaceus. Aquaculture. 2006; 259: 432–443. 10.1016/j.aquaculture.2006.05.021

[26] Li LQ, Wang D, Liu XY, Zhang B, Liu YZ, Xie T, et al Soil organic carbon fractions and microbial community and functions under changes in vegetation: a case of vegetation succession in karst forest. Environ Earth Sci. 2014; 71(8): 3727–3735. 10.1007/s12665-013-2767-3

[27] Fuhrman JA, Cram JA, Needham DM. Marine microbial community dynamics and their ecological interpretation. Nat Rev Microbiol. 2015; 13: 133–146. 10.1038/nrmicro3417 25659323

[28] Gilbert JA, Field D, Swift P, Newbold L, Oliver A, Smyth T, et al The seasonal structure of microbial communities in the Western English Channel. Environ Microbiol. 2009; 11: 3132–3139. 10.1111/j.1462-2920.2009.02017.x 19659500

[29] Tinta T, Vojvoda J, Mozetič P, Talaber I, Vodopivec M, Malfatti F, et al Bacterial community shift is induced by dynamic environmental parameters in a changing coastal ecosystem (northern Adriatic, northeastern Mediterranean Sea) a 2-year time-series study. Environ Microbiol. 2015; 17: 3581–3596. 10.1111/1462-2920.12519 24903068

[30] Liu GH, Rajendran N, Amemiya T, Itoh K. Bacterial community structure analysis of sediment in the Sagami River, Japan using a rapid approach based on two-dimensional DNA gel electrophoresis mapping with selective primer pairs. Environ Monit Assess. 2011; 182: 187–195. 10.1007/s10661-010-1868-7 21222030

[31] Martins G, Henriques I, Ribeiro DC, Correia ACM, Bodelier PLE, Cruz JV, et al Bacterial diversity and geochemical profiles in sediments from eutrophic Azorean lakes. Geomicrobiol J. 2012; 29: 704–715. 10.1080/01490451.2011.619633

[32] Cheng W, Zhang J, Wang Z, Wang M, Xie S. Bacterial communities in sediments of a drinking water reservoir. Ann Microbiol. 2014; 64: 875–878. 10.1007/s13213-013-0712-z

[33] Castine SA, Bourne DG, Trott LA, McKinnon DA. Sediment microbial community analysis: establishing impacts of aquaculture on a tropical mangrove ecosystem. Aquaculture. 2009; 297: 91–98. 10.1016/j.aquaculture.2009.09.013

[34] Kawahara N, Shigematsu K, Miura S, Miyadai T, Kondo R. Distribution of sulfate-reducing bacteria in fish farm sediments on the coast of southern Fukui Prefecture, Japan. Plankton Benthos Res. 2008; 3: 42–45. 10.3800/pbr.3.42

[35] Asami H, Aida M, Watanabe K. Accelerated sulfur cycle in coastal marine sediment beneath areas of intensive shellfish aquaculture. Appl Environ Microb. 2005; 71: 2925–2933. 10.1128/AEM.71.6.2925–2933.2005 PMC1151846 15932986

[36] Bissett A, Bowman J, Burke C. Bacterial diversity in organically-enriched fish farm sediments. Fems Microbiol Ecol. 2006; 55: 48–56. 10.1111/j.1574-6941.2005.00012.x 16420614

[37] Srithep P, Khinthong B, Chodanon T, Powtongsook S, Pungrasmi W, Limpiyakorn T. Communities of ammonia-oxidizing bacteria, ammonia- oxidizing archaea and nitrite-oxidizing bacteria in shrimp ponds. Ann Microbiol. 2015; 65: 267–278. 10.1007/s13213-014-0858-3

[38] Fukui Y, Abe M, Kobayashi M, Saito H, Oikawa H, Yano Y, et al Polaribacter porphyrae sp. nov., isolated from the red alga Porphyra yezoensis, and emended descriptions of the genus Polaribacter and two Polaribacter species. Int J Syst Evol Micr. 2013; 63: 1665–1672. 10.1099/ijs.0.041434–0 22904227

[39] Sakai T, Kimura H, Kato I. A marine strain of Flavobacteriaceae utilizes brown seaweed fucoidan. Mar Biotechnol. 2002; 4: 399–405. 10.1007/s10126-002-0032-y 14961251

[40] Mark JK, Benjamin BC, Jevon JH, Oyenike OO, Alessandra CL, Satish CBM, et al Natural niche for organohaliderespiring Chloroflexi. Appl Environ Microb. 2012; 78: 393–401. 10.1128/AEM.06510-11 22101035 PMC3255752

[41] Fan LM, Barry K, Hu GD, Meng Sl, Song C, Wu W, et al Bacterioplankton community analysis in tilapia ponds by Illumina high-throughput sequencing. World J Microb Biot. 2016; 32: 10 10.1007/s11274-015-1962-7 26712625

